# The roots of trauma: a review of the history of psychotrauma

**DOI:** 10.1590/S0104-59702023000100039

**Published:** 2023-08-11

**Authors:** Ramon Reis, Francisco Ortega

**Affiliations:** i Professor substituto, Departamento de Saúde Mental/Faculdade de Medicina/Universidade Federal do Rio de Janeiro. Macaé – RJ – Brasil ramonrsferreira@gmail.com; ii Professor pesquisador, Catalan Institution for Research and Advanced Studies. Barcelona – Catalunha – Espanha. Professor, Medical Anthropology Research Center/Universitat Rovira i Virgili.Tarragona – Catalunha – Espanha francisco.ortega@icrea.cat; Medical Anthropology Research Center, Universitat Rovira i Virgili, Tarragona, Catalunha, Espanha, francisco.ortega@icrea.cat

**Keywords:** Psychological trauma, History, Health, Post-traumatic stress disorder (PTSD, Neuroscience, Trauma psicológico, História, Saúde, Transtorno de estresse pós-traumático (TEPT, Neurociências

## Abstract

Perceptions of the importance of the role of psychological trauma in the origins of psychiatric problems have oscillated throughout the history of psychiatry. However, since the conception of post-traumatic stress disorder (PTSD), western societies have witnessed a marked expansion of the discourse of trauma in the interpretation of devastating human experiences like catastrophes, genocides, disasters, and epidemics. Through an integrative literature review, this article analyzes some of the historical and epistemological determinants behind the emergence of traumatic memory and the establishment of trauma as a semantic field that orients clinical responses and political strategies in the field of the humanities and the health sciences.

## Resumo

A percepção do papel do trauma psicológico na origem de problemas psiquiátricos aumentou e diminuiu ao longo da história da psiquiatria. Com a concepção do transtorno de estresse pós-traumático (TEPT), entretanto, as sociedades ocidentais presenciaram uma profunda expansão do discurso do traumatismo na interpretação de experiências humanas devastadoras, como catástrofes, genocídios, desastres e epidemias. A partir de revisão bibliográfica integrativa, este artigo analisa alguns dos determinantes históricos e epistemológicos que fundamentam o surgimento da memória traumática e o estabelecimento do trauma como campo semântico que orienta respostas clínicas e estratégias políticas no campo das ciências humanas e da saúde.

Especula-se que um trauma, em qualquer dimensão existencial na qual incida, seja capaz de transformar profundamente um determinado estado de coisas (van der Kolk, 2002). Muito embora uma definição unívoca para o trauma pareça ainda distante no campo da psicotraumatologia e na pesquisa neurobiológica do estresse traumático (Yehuda, 2004), é inegável que sua denominação contemporânea está extensivamente atrelada a eventos potencialmente capazes de produzir uma conjuntura específica pela ação de seus efeitos avassaladores (van der Kolk, McFarlane, 1996). Quando uma catástrofe natural assola um determinado grupo humano, quando um abuso sexual explicita os seus efeitos deletérios sobre o psiquismo individual de uma vítima, ou mesmo quando a ação vulnerante de agentes mecânicos efetiva uma lesão acidental do tecido ósseo, o que está em questão é o discurso do traumatismo – uma linguagem que se presume ter nascido no crepúsculo do século XVII. Desde 1980, quando, por ocasião do lançamento da terceira edição do *Manual diagnóstico e estatístico de transtornos mentais* (DSM-III), a American Psychiatric Association (APA) inclui o transtorno de estresse pós-traumático (TEPT) em sua nosografia oficial (American..., 1980), essa linguagem passa a ser recrutada por toda a sorte de aparatos políticos, clínicos e humanitários que direcionaram respostas de enfrentamento para os processos de traumatização (Theidon, 2013; Griffin, 2020).

A partir de então, não é incomum que desastres de larga escala, como os ocorridos nas cidades de Mariana e Brumadinho (MG), e calamidades de proporções globais, como a recente pandemia da covid-19, sejam examinados à luz de uma mesma matriz interpretativa – aquela que estabelece uma causalidade discernível entre fenômeno perturbador e sintoma correspondente, e a qual Allan Young (1996a) resumiu sob a concepção de uma nova retórica do sofrimento (Demertzis, Eyerman, 2020; Reis, Ortega, 2021). Daí resultará a elaboração de critérios de inclusão e exclusão dedicados, na esfera público-política, a inventariar eventos, episódios e contextos sociais, segundo o seu potencial perturbador – uma classificação sub-reptícia do domínio do traumático (Hinton, Lewis-Fernández, 2010; Hinton, Good, 2016).

Com a expansão dos diagnósticos psiquiátricos conquistada após a revolução nosográfica que "canonizou" os fundamentos biológicos dessa disciplina (Brody, 1990), o TEPT adquire o estatuto formal de porta-voz e representante maior dos padecimentos secundários a um trauma (Kirmayer, Lemelson, Barad, 2007).

A propagação exponencial da entidade mórbida sugere, por consequência, que a racionalidade ocidental contemporânea estaria de acordo com a ideia de que a subjetividade está sujeita às mesmas consequências que permitem avariar o corpo biológico. O entrelaçamento das tradições psicológica e somática da pesquisa do trauma, documentada nas historiografias de Allan Young (1995) e Fassin e Rechtman (2009) sob as respectivas formas de uma genealogia científica e moral, destaca o nascimento da especulação acerca de uma subjetividade passível de traumatização e do trauma como memória expressa de uma violência passada (Kirmayer, Lemelson, Barad, 2007; Bistoen, 2016).

Esse é um momento decisivo para a história dos estudos sobre o traumatismo – momento em que o sofrimento mental é anexado ao domínio do traumático, o que corresponde, virtualmente, ao momento exato da fundação de um discurso do traumatismo cuja herança se confunde com a própria economia moral contemporânea (Fassin, Rechtman, 2009). A possibilidade de conversão da violência em dano traumático marca a inauguração do compromisso fenômeno-sintoma no domínio da causalidade traumática, porém ainda mais especialmente determina a própria liberação da racionalidade ocidental para a admissão da capacidade de um evento ser o provocador de uma modificação vindoura na montagem subjetiva de um indivíduo (van der Kolk, McFarlane, 1996).

Da primeira menção ao verbete "traumático", localizada na versão de 1656 do *Oxford english dictionary*, até o seu ressurgimento na psiquiatria do século XIX, a terminologia do trauma migra, pois, de uma significação restrita às lesões mecânicas da medicina cirúrgica, para ser posteriormente readmitida como metáfora plausível na representação de estados psicológicos adversos com contornos etiológicos ainda imprecisos. A subjetividade traumatizada presume, portanto, esse deslocamento epistemológico que extrapolou a significação do termo "trauma" da substância *extensa* ao *cogito* (Descartes, 1973), e pelo qual uma inédita perspectiva semântica da traumatização derivou uma nova espécie de memória: a memória traumática (Young, 1995).

Com a atomização da unidade do trauma em subtipos traumáticos múltiplos, a contemporaneidade testemunhou a versatilidade que a transformação do conceito em narrativa proporcionou à expansão de seu domínio; para cada evento penoso, um modo específico de padecimento (Marsella et al., 2008); para cada ocorrência danosa, um tipo particular de trauma. Acidentes automobilísticos, assaltos, surtos epidêmicos, enfim, toda a sorte de misérias sociais pôde ser codificada sob a rubrica do psicotraumatismo. Sendo um conceito interdisciplinar, ele se presta, no campo da saúde pública, a explicar a origem e a prevalência de disparidades étnico-raciais de saúde resultantes do trauma de massa (Sotero, 2006); na arena das ciências sociais, por seu turno, ele permite a extrapolação da sua espessura conceitual para justificar que uma dimensão trágica da existência humana reside em todas as formas de aprofundamento da precarização da vida (Wilkinson, Kleinman, 2016).

Este artigo aborda alguns dos determinantes históricos e epistemológicos que fundamentam a racionalidade contemporânea do psicotraumatismo, expressa essencialmente no estabelecimento do trauma como campo semântico e na memória traumática como um representante da ordenação desse edifício discursivo. Inicialmente, destacamos que o surgimento do termo "trauma" aplicado às lesões corporais identificadas pela medicina cirúrgica do século XVII origina uma memória traumática do corpo. Em paralelo, procuramos demonstrar como a interpretação psicodinâmica do sonambulismo e dos fenômenos amnésicos no século XIX culmina na constituição de uma memória traumática psicologizada. Por fim, analisamos o intercâmbio dessas duas modalidades do trauma seguindo a proposta genealógica de Allan Young (1995, 1996a, 1996b) e sugerimos que as transformações no modo de conceber a psicobiologia dos estados mórbidos, iniciada há três séculos, autoriza uma leitura transdisciplinar das consequências danosas de estressores extremos.

Espera-se que este trabalho contribua para uma análise crítica acerca da maneira como essa discursividade operou classificações específicas da realidade, hierarquizando experiências radicais, segundo diferentes tipologias traumáticas (desastres naturais, guerras, genocídios, casos de violência doméstica, terrorismos etc.), e condicionadas a respectivos estressores particulares (lesões físicas ou psicológicas, por exemplo).

## A memória traumática do corpo

Diversos autores defendem que a descoberta e a invenção da memória traumática tenham mediado, estabilizado, e, finalmente, concluído a transição da traumatização do corpo para a mente (Hacking, 1994, 1996; Young, 1995, 1996a; Fassin, Rechtman, 2009; van der Kolk, 2016). Essa alegação sintetiza uma parcela verdadeira do processo que culminará na redescoberta do estresse traumático no século XX, e é justificável que dois desenvolvimentos tenham sido centrais para a unificação da patogenia psíquica e anatômica no paradigma do traumatismo: a emergência de uma concepção universalista do corpo e da dor física; e o reconhecimento da natureza diversa da memória. Os múltiplos entrecruzamentos dessas duas correntes – somática e psicológica – da investigação científica originam o que se conhece atualmente por "memória traumática" (Young, 1995). De acordo com Sanfelippo (2018), entretanto, a trajetória até a "psicologização do trauma", iniciada no século XIX, é rigorosamente a história de uma transformação gradual no reconhecimento das condições pós-traumáticas pela adição de significações inéditas à descrição médico-cirúrgica (anterior), e, por essa razão, seriam imprecisas as tentativas de descrevê-la por alusão a um "salto" ou "corte" epistemológico inaugurados em disciplinas ou saberes específicos.

Para que a noção de trauma adquirisse um significado psicológico, determinadas transformações no saber médico teriam sido necessárias, tais como a construção de corpos heterogêneos, ou a delimitação de novas fronteiras entre a psicologia e a medicina, entre o somático e o psíquico. A transformação na noção de trauma não segue etapas bem definidas, mas responde também por processos culturais e sociais extradisciplinares e por transformações epistêmicas no conhecimento médico (independentes e anteriores ao problema específico do trauma). Assim, a análise da mutação intrínseca ao discurso médico do século XIX ajudaria a revelar os elos intermediários entre as versões completamente somáticas ou as completamente psicológicas da noção de trauma.

A historiografia do trauma psicológico aponta para a obra de John Erichsen (1867) como a coordenada inicial da trajetória da memória traumática (Trimble, 1985; Young, 1996a; van der Kolk, 1996; Matus, 2001). Nela, Erichsen (1867, p.xi) analisou certas "lesões obscuras do sistema nervoso" frequentemente encontradas como resultado do choque mecânico ocasionado por colisões ferroviárias. Incentivando a percepção de que mudanças moleculares invisíveis e não detectadas no sistema nervoso, no cérebro e na coluna vertebral seriam responsáveis por paralisia, tremores ou perda dos sentidos, o trabalho de Erichsen transformou-se numa referência-padrão na literatura médica e jurídica em um ambiente social convulsionado por reivindicações das classes trabalhadoras acerca da precariedade das condições do tráfego ferroviário (Leese, 2002). A classificação por ele proposta agrupava casos clínicos oriundos de acidentes ferroviários em três categorias – aqueles em que fortes golpes ou "choques" teriam danificado o tecido neural visivelmente ao exame *post mortem*; aqueles em que a sintomatologia seria consequência de golpes, tremores ou trepidações apenas triviais; e, por fim, os casos supostamente simulados como meio de subtrair compensações financeiras aos emergentes sistemas de seguridade social do século XIX (Young, 1995).

O argumento de Erichsen é original em diversos sentidos: primeiro, porque esboça uma tentativa de solução para o "choque" – fenômeno enigmático para a medicina cirúrgica do século XIX, definido por Edwin Morris (1867, p.9) como "o efeito peculiar sobre os sistemas animais, produzido por ferimentos violentos de qualquer causa ou por emoções mentais violentas – como tristeza, medo, horror ou repulsa"; segundo, porque institui uma racionalidade traumática alinhada aos preceitos da traumatização psicogênica, abrindo caminhos para que as nosografias psiquiátrica e psicológica revolucionassem a compreensão dos distúrbios pós-traumáticos; e, finalmente, porque materializa os anseios de uma época em explicar a inusitada incongruência entre a gravidade de uma lesão e a gravidade de um sintoma, unificando, dessa forma, um arco abrangente de sofrimentos em torno de mecanismos causais análogos (Amir, Kaplan, Kotler, 1996; Ogle, Rubin e Siegler, 2013).

A obra de Erichsen (1867) se inscreve, portanto, em uma "zona cinzenta" entre a lesão verificável do sistema nervoso e a simulação. No interior dessa "zona", começam a ser testadas novas explicações sobre a patologia nervosa que acabaram resultando na construção de novas acepções para o termo médico do trauma (Sanfelippo, 2018). Antes, seria improvável a atribuição de paralisias, dormências, estados depressivos, hipervigilância, insônia, pesadelos, amnésia e outros fenômenos secundários na ausência de uma lesão física aparente, o que explica a dificuldade em estabelecer uma relação causal entre o acidente e o aparecimento retardado dos sintomas. Desde o advento da categoria da *railway spine*, portanto, a gravidade do acidente não é mais suficiente para efetivar a determinação etiológica, pois seria o choque – e não a lesão mecânica – o responsável por danificar o sistema nervoso em graus mais ou menos sutis.

Para Young (1995), a própria ideia de que o efeito de uma violência infligida em uma parte do organismo pode ser transmitido e reespacializado em outras partes pressupõe a existência de uma estrutura anatômica capaz de alinhavar todas as parcelas do corpo. Uma vez que o choque pode ocorrer na ausência de lesões ou hemorragias visíveis, coube a Erichsen inferir um elemento etiológico exclusivo, capaz de organizar, em uma mesma superfície psicopatológica, tanto os sintomas de pessoas acometidas por formas brandas de choque nervoso quanto os de pessoas sujeitas a formas graves de concussão espinhal e de choque cirúrgico (Meumark, 1947). Esse elemento foi o medo.

Com o medo, temos o nascimento de uma psicologia geral do choque capaz de elucidar o mecanismo de tradução das consequências de um trauma severo pela ação solitária de uma emoção. Rigorosamente equiparada a um golpe ou lesão física, a emoção passa a chancelar, de forma inédita até o século XIX, uma modalidade explicativa alheia à dimensão orgânica na presunção etiológica de distúrbios secundários a choques traumáticos (Young, 1996b). É por essa razão que a chamada "inconsciência mental ou moral" (Harrington, 2003) poderia ocorrer sem a imposição de qualquer lesão física, golpe ou agressão direta na cabeça ou na coluna, mas pela mera exposição a graus comparativamente insignificantes de violência (Erichsen, 1867). O acontecimento súbito, a ausência de tempo de preparação para o choque e a impotência seriam, logo, aquilo que comumente captura as vítimas de colisões ferroviárias em uma vivência de "terror frenético" (p.196) supostamente responsável pelo desfecho traumático. O choque é, portanto, uma noção central na mudança da abordagem materialista para uma abordagem psicológica da vida mental (Tager, 1973).

> De repente, fez sentido para os médicos enfatizar a fenomenologia do choque cirúrgico, combinando anestesia com ambientes hospitalares aprimorados. Na Primeira Guerra Mundial, o debate sobre a *railway spine* reapareceu em relação a soldados em choque, mas aparentemente ilesos; sugestões de concussão espinhal (apoiadas por lâminas de fluido espinhal ‘localizando’ o ferimento) deram lugar, com o progresso da guerra, à terapia (Armstrong, 2000, p.61).

Poucos anos transcorreram até que o medo, a lesão física e as variadas modalidades de choque fossem agrupadas em torno de uma experiência ainda mais originária. A experiência da dor – postulado representativo de um conhecimento corporal que, ao enraizar a lesão, prenuncia coincidentemente a mortalidade (Young, 1996a) – adquire uma silhueta evolucionária e é convertida em parâmetro essencial da sobrevivência de toda uma espécie, pois, mesmo que os organismos não devam temer a dor em si mesma, eles devem evitar a lesão que a acompanha (Bonavita, De Simone, 2011). A partir de então, se uma contusão grave, uma doença infecciosa ou mesmo circunstâncias inócuas “impulsionam o sistema cinético a uma velocidade avassaladora” (Crile, Lower, 1920, p.264), desencadeando então a patologia essencial do choque, é porque a dor, reconhecida na extremidade da cadeia de eventos que conduz à lesão, passou a servir de arquétipo para a unificação da experiência do choque, sejam quais forem as suas causas (Armstrong, 2000). Evitando a dor, os organismos evitariam, então, a lesão e as suas consequências obrigatórias, expressas sob as figuras radicais do prejuízo funcional e da destruição da espécie (Bonavita, De Simone, 2011). É por haver ascendência entre uma disposição corporal específica e uma reminiscência correlata, que o medo é considerado a própria memória da dor (Spencer, 1855); e para Herbert Spencer, seu visionário, o “elo perdido na genealogia da memória traumática” (Young, 1996b, p.253).

## Dupla genealogia

O século XIX presencia, dessa maneira, a conversão da memória em polo de integração da experiência traumática, viabilizando o mútuo deslizamento da traumatização do corpo para o território da mente e deste para aquele. Ian Hacking (1994, 1996) sinalizou que o pressuposto da universalidade do corpo e da dor física, originariamente mapeado nos teatros cirúrgicos e nos experimentos com animais em laboratórios de fisiologia, forneceu à ciência e à medicina uma grande parcela de autoridade para falar a respeito do sofrimento traumático da mente. No entanto, os efeitos primários e os resultados secundários de lesões leves no sistema nervoso (Erichsen, 1867) não esgotam as diversas modalidades de apreensão regular do fenômeno do sofrimento, e, ao reivindicar a precedência do choque cirúrgico como um dos precursores da memória traumática, ou assumir que o pensamento de Erichsen tenha efetivamente autonomizado a sintomatologia traumática de suas causas materiais objetivas, confina-se o debate da traumatização a apenas uma das suas ramificações ontológicas.

A literatura psiquiátrica adota geralmente a tese de que o trauma cirúrgico do século XVII teria sido convertido em trauma psíquico nas disciplinas psicodinâmicas ao final do século XIX (van der Kolk, 2002). Uma transferência por analogia teria distendido o enunciado da traumatização mecânica até os domínios obscuros do psiquismo, concretizando, a um só

tempo, a restauração do trauma em um renovado campo semântico e, sobretudo, a uma abertura indefinida da sua circunscrição (Young, 1995; Leys, 2000, 2006; Leese, 2002; van der Kolk, 2002, 2016). A metáfora do trauma teria sido transposta de um plano a outro – do mecânico para o psíquico, do material para o abstrato, do corpo para a mente. Por meio dela, fundiram-se os palcos físico e psicológico, e o trauma passaria a percorrer a superfície extensa, quase infinita, de uma malha de articulações de sentido. Entretanto, a hipótese da transferência por analogia foi frontalmente contestada por Young (1995, 1996a), para quem essa perspectiva, embora historicamente comum, é imprecisa, pois os dois tipos de trauma – o orgânico e o psicológico/existencial – estariam vinculados por genealogia, não por semelhança. Suas respectivas memórias, independentemente da apresentação somática ou psicológica pela qual se expressem, representam, ainda assim, versões de uma matriz traumática comum.

Ao argumentar que a concepção de memória não participava essencialmente do processo patogênico na perspectiva somática da traumatização, Sanfelippo e Dagfal (2020) parecem divergir das hipóteses de Young (1995) para a história do trauma psicológico. Para este, a memória jamais teria sido desconsiderada na patogênese, embora o seu lócus tenha sido posicionado diferentemente na tradição médica e psicológica da pesquisa do trauma. Todos concordam, porém, que processo de memorialização do trauma coincide com o processo de psicologização do trauma, uma vez que a memória traumática é condição indispensável a qualquer concepção de trauma subjetivo (Young, 2004).

Por um lado, a memória traumática encarnada, como produto mediado das relações históricas que a dor estabeleceu com o medo, equivale em sua natureza ao conjunto de relações internas absolutas e potencialmente presentes antes do nascimento, talvez sob a forma de conexões nervosas definitivas (Young, 1996a). É claro que cada memória filogenética começa com uma experiência individual, e, nesse sentido arcaico, ela remete à sua origem – fundamentalmente ao indivíduo. No entanto, quando o traço neurológico deixado na estrutura do sistema nervoso transcende em absoluto a experiência individual pela reprodução de relações internas predeterminadas, a profundidade desse registro orgânico independente unifica a vivência acumulada de múltiplas gerações. É assim que a memória corporal retrocederá à memória coletiva, e esta será revertida em instinto. A memória se torna instinto porque, liberada da atividade cognitiva e inscrita vagarosamente no tecido neuronal, ela representará uma espécie de cicatriz – o resultado do corte que a experiência esculpiu no corpo com a cooperação imprescindível do tempo e das gerações.

À medida que o medo se torna a memória da dor – e pudemos alcançar tal conjectura pela associação de suas disposições constitucionais (Spencer, 1855), da biologia evolucionária (Darwin, 1965) e da unificação da experiência dos choques (Crile, 1899) –, temos cumpridas as exigências mínimas ao desenvolvimento de uma teoria coerente da memória traumática de natureza somática. A memória filogenética de Spencer recapitula a história evolucionária de uma espécie no desenvolvimento embrionário de seu membro individual (Hacking, 1994). A ideia-chave de que o medo e a raiva seriam expressões duais de um mesmo fenômeno fisiológico, originado em seu pensamento, possibilitou que o medo, como memória da dor, olhasse para o passado, mas igualmente para o futuro, sob a forma de ações de luta e fuga que permitiriam ao organismo prevalecer ao evitar a dor, a lesão e, por fim, a morte. Ao corpo foi dito para se lembrar da dor, e "o 'medo' foi o nome dado a essa memória" (Young, 1996a, p.260), pois o medo e a dor foram ambos transmutados na qualidade de herança evolutiva habilitada a antecipar ameaças e a evitar a destruição. A dor e o medo foram, portanto, normalizados, transformados em memória, e a inabilidade para experimentar a dor passa a ser a representação de um tipo perigoso de ignorância.

O deslocamento da dor e a espacialização do medo anunciam uma experiência biopsicossocial humana caracterizada pela transcrição bilateral das emoções no corpo e dos sofrimentos do corpo nas emoções, cujo resultado parece ter produzido um tipo de memória rebelde às imagens, sensações e emoções constituintes do espaço psicológico: uma memória mais simples, enraizada na neuroanatomia, na fisiologia e na história evolucionária (Young, 1996b). Com a incorporação dos fenômenos psicológicos ao monopólio da pesquisa neurocientífica, passaremos a ter notícias de uma memória traumática corporal bloqueada nos sistemas límbico e simpático, passível de ser revisada apenas por meio de mecanismos evolutivos (van der Kolk, 2016).

## O trauma (psíquico) da memória

Por outro lado, uma segunda cadeia de eventos nasce no final do século XVIII, na interseção entre as investigações do sonambulismo e as posteriores especulações acerca dos distúrbios amnésicos de natureza psicogênica. As primeiras descrições de distúrbios pós-traumáticos impulsionados pela memória datam das décadas de 1870 e 1880, e referem-se a casos envolvendo sintomas pseudoneurológicos (Young, Breslau, 2016). Duas décadas após os trabalhos de Erichsen com o choque e o medo terem fixado a compreensão moderna do trauma, Jean-Martin Charcot abre o caminho irreversível para uma abordagem psiquiátrica desse fenômeno (Fassin, Rechtman, 2009). Efetivando o medo como fator patogênico e a neuromimese como dispositivo etiológico para uma abordagem psicológica da *railway spine*, Charcot (1889) postulou que os efeitos da síndrome se seguiriam a um estado hipnótico autoinduzido, quando estão mais ou menos rebaixados a espontaneidade mental, a vontade ou o julgamento. Por meio de neuromimese, a mais insignificante lesão no braço ou na perna experimentada em estado hipnótico seria transformada em paralisia ou contratura. A obra de Charcot simboliza uma virada epistemológica representativa da atribuição de condições pós-traumáticas a certas disposições afetivas potenciais – algo já insinuado desde a hipótese da interveniência do medo sobre a causação do choque em Herbert Page (1883), embora ainda lhe faltasse conhecimentos sobre os mecanismos etiológicos específicos.

Com Charcot, entrecruzam-se as duas trajetórias científicas da perspectiva moderna do trauma: "A linha que leva de Erichsen a Crile, Cannon e Pavlov, traçando a evolução da memória traumática somatizada; e a linha que leva de Charcot a Janet e Freud, traçando a evolução de uma memória traumática psicologizada" (Young, 1995, p.268). Naquela época, o debate da traumatização consistia no tensionamento entre ambas as perspectivas epistemológicas e na elaboração de entidades psicopatológicas dispersas e de semiologias caóticas (Oppenheim, 1889; Leese, 2002). Uma importante tentativa de sistematização do campo foi formalizada por Hermann Oppenheim (1889), que isolou a fenomenologia da *railway spine* em uma entidade mórbida completa – a neurose traumática –, e cuja implementação indicava a pregnância determinante do paradigma neurológico sobre a compreensão das afecções traumáticas.

O conceito de neurose traumática localizava os sintomas nervosos pós-traumáticos entre as afecções histérica e neurastênica, considerando-os simultaneamente a consequência de reações físicas ao susto e a causa de alterações do tecido molecular (Holdorff, Dening, 2011). Para Charcot, entretanto, a proposta de reestruturação semiológica não representava benefícios significativos à compreensão dos distúrbios secundários ao trauma, pois as suas principais características – paralisias, contraturas, anestesias, alterações sensoriais e do humor – seriam indiscerníveis dos sintomas encontrados em quadros histéricos, e a sua origem particular, insuficiente para realizar o diagnóstico diferencial (Young, 1995). O medo, o terror e o susto – fatores etiológicos distintivos das pretensas neuroses traumáticas (Oppenheim, 1889) – seriam, portanto, incapazes de consolidar uma categoria psicopatológica unívoca e desmembrada das demais síndromes constituintes do espectro do traumatismo (Micale, Lerner, 2001).

Erichsen (1867) é, portanto, quem entrelaça os choques cirúrgico e nervoso à mesma fonte patoanatômica, e Page (1883) quem os dispõe em estruturas neurológicas paralelas, mas independentes. Porém, é somente com Charcot que o discurso do trauma purifica as condições pós-traumáticas de suas referências ao tecido nervoso e às estruturas neurais, abrindo caminho para a territorialização do aparato psíquico, com a sua complexa demarcação topográfica e com as enigmáticas leis que regem o seu funcionamento. Se o choque cirúrgico e, por ingerência da dor, igualmente o choque nervoso presidiam a efetivação do traumatismo, é justamente porque, para a perspectiva somática dos distúrbios traumáticos, o trauma é apenas um produto da fisiologia do choque. Em sua matriz psicológica, todavia, o trauma passa a ser concebido como vivência de imitação ou identificação hipnótica (Leys, 2000); e o sintoma oriundo do traumatismo psíquico se converte em simulacro daquele que provém da lesão orgânica. Esse paradigma da mimese corporifica uma compreensão "fisiológica" da mente que preside a difusão do modelo da histeria como protótipo da psicologização da memória traumática na virada para o século XX.

Qualquer que fosse a causa desencadeante – traumatismo, intoxicação, terror ou frustração amorosa –, a histeria aproximava as descrições fenomênicas do choque cirúrgico e do choque nervoso, interrogando as fronteiras da patognomia histérica (Micale, Lerner, 2001). Mascarada sob outro nome qualquer – e, eventualmente, mesmo "imitando uma doença orgânica legítima" (Fisher, 1913, p.414) –, ela representava a máxima escansão do gesto diagnóstico, a transição ilimitada por meio da qual uma entorse ou uma fratura nas costas seriam reclassificadas como histeria pura. Essa correção contínua à que progredia e regressava indefinidamente o raciocínio clínico criou uma oportunidade para que a histeria pudesse ser uma consequência igualmente possível da consecução traumática, uma sequela final passível de condensar todos os signos da traumatização.

Os meios pelos quais a dor e o medo confluem para o choque nervoso e, em paralelo, a emoção violenta aciona a disposição histérica são os desenvolvimentos que refletem a fusão dessa dupla trajetória que vai da criação de uma memória evolutiva desprovida de palavras e de imagens e, percorrendo os interstícios do segredo patogênico, finda por arrematar os perfis visíveis da memória traumática (Young, 1995).

## A doutrina do esquecimento

Allan Young (1995) argumenta que medicina do final do século XIX teria inventado uma concepção de memória estranha às suas denotações tradicionais de proficiência cognitiva, à faculdade de estocar informações ou mesmo à recuperação de um evento passado. A quarta dobra da memória, cuja significação remonta ao efeito patogênico da guarda de um segredo perturbador sobre aquele que o porta – e igualmente a ação curativa atribuída à sua confissão –, recebeu o nome de "segredo patogênico" (Ellenberger, 1966) e contribuiu notavelmente para a origem da psiquiatria dinâmica moderna.

Frequentemente, o segredo patogênico assume o aspecto de uma neurose crônica que, "de acordo com o lugar e a época, pode ser chamada de melancolia, neurastenia, histeria ou mesmo psicose" (Ellenberger, 1966, p.29). É igualmente suposto de ser o responsável, em suas expressões mais drásticas, por interrupções súbitas da vida humana ou pelas formas menos incomuns do comportamento irracional ou extravagante (Leys, 2000). Ao ocultar uma paixão reprimida, uma doença orgânica ou um desvio moral (Ellenberger, 1966), o segredo patogênico apresenta toda a sua amplitude; o ciúme, o ódio, a ambição, uma característica particular da qual se envergonhe o sujeito ou mesmo a dolorosa lembrança de um acontecimento traumático podem fabricá-lo. Em todos os casos, o panorama subjetivo é decisivo: "O que realmente importa não é tanto o fato em si, mas o significado que o paciente atribui a ele de acordo com sua escala pessoal de valores" (p.29).

Se o segredo patogênico indica que uma determinada memória seria capaz de prosseguir afetando o comportamento de um sujeito, pois fora convertida em fonte de sofrimento, a própria memória traumática representaria um subgrupo desse fenômeno – ela é uma classe de segredo patogênico (Young, 1995). "Tais memórias são 'patogênicas' porque a elas é reputada a causa de transtornos psiquiátricos – a histeria, no final do século XIX, e o TEPT, no final do século XX –, e são um 'segredo' porque representam um ato de encobrimento" (Young, 1995, p.369). Em sua vasta história desde as primeiras ocorrências em civilizações antigas, quando se o presumia estar em relação de direta causalidade com o tabu e o pecado, o segredo patogênico conserva a sua característica de denunciar a impotência e a desesperança (Ellenberger, 1966). A memória que esse segredo encobre é o registro mental da própria experiência traumática (Young, 1996b) sujeita aos mais diversos engenhos e deformações, como confirmam a dissociação, no pensamento de Janet (1889), ou o recalque, na teoria psicanalítica (Freud, 2016, 2010b). A diferença entre a memória ordinária e o segredo patogênico é clara: a primeira constitui um traço mnêmico; a segunda, um parasita mental.

Essa perspectiva do segredo se articula com uma concepção central do esquecimento no discurso da traumatização psicogênica, ao evidenciar os aspectos da memória que pareciam particularmente superiores aos poderes humanos de "recordar e esquecer" e que tanto fascinaram o pensamento vitoriano em sua procura obstinada pela regulamentação das operações inconscientes do psiquismo (James, 1894; Freud, 2019). É verdadeiro que, desde o princípio, a obra de Freud enfatizou os efeitos do choque sobre a memória. Entretanto, ainda que a partir da metade do século XIX se verifiquem tratados médicos destinados a descrever os choques e as lesões originadas de acidentes ferroviários, pouca atenção foi concedida aos efeitos dos choques traumáticos sobre os processos de consolidação e de recuperação da memória. O que está em questão com esse fenômeno é justamente a latência a que se parecia sujeitar a memória em circunstâncias em que o choque foi requisitado como fator etiológico do traumatismo subsequente. Uma vez que a memória passou a prefigurar as condições de causação do estado mórbido, tornou-se necessário compreender o que ocorre entre os intervalos de sua ativação, isto é, por que meios a memória se oculta após um acontecimento potencialmente avassalador e como, de súbito, ela logra ressurgir.

No último quarto do século XIX, a noção de que uma ideia poderia ser reproduzida por meio de ação sugestiva, por mais longo que fosse o intervalo, já era geralmente aceita na psicologia. Pelo mesmo princípio, qualquer outro estado de consciência ao qual aquela ideia estivesse originalmente associada poderia recorrer de sua condição "dormente" ou "latente", desde que atingisse as condições apropriadas para o ingresso na esfera da consciência (Carpenter, 1876). Essa concepção positiva do esquecimento é cardinal para a compreensão do desenvolvimento que a memória traumática mentalizada/psicologizada percorreu entre a sua emergência, no solo inicial da amnésia durante o século XIX, e a sua instalação definitiva sobre a ideia da sugestão pós-hipnótica, quando foi transmitida finalmente à psiquiatria do século XX.

Ao veicular inexoravelmente a ferida médica à ferida psicológica, o par memória/esquecimento extraiu o trauma da literatura especializada e o situou como metáfora abrangente no conhecimento popular. De acordo com Young (1995), o uso cotidiano do termo memória guarda três acepções principais: "A capacidade mental de reter informação adquirida e realizar operações aprendidas; o conteúdo semântico, imagético ou sensório das lembranças; e a localização onde essas lembranças são armazenadas" (Young, 1995, p.42-43). Se, como supunham Locke e Hume, a memória estaria intrinsicamente conectada à nossa concepção de *self* e de autoconsciência, fornecendo ao corpo um sujeito e uma subjetividade, podemos conjecturar que a invenção da memória traumática constitua o eixo da revolução que conduziu a sensibilidade moderna a alicerçar a formação do caráter e da personalidade individuais não mais sobre aquilo de que se lembra, mas daquilo de que se esquece (Hacking, 1994; Young, 1995).

Essa "doutrina do esquecimento" (Hacking, 1996, p.67) e, por sua vez, também o trauma psíquico transformaram a própria concepção de um Eu espiritual (alma) no Ocidente, operando dois desenvolvimentos axiomáticos: a revisão dos atributos centrais do *self* ocidental – o livre-arbítrio e o autoconhecimento (a capacidade de refletir e efetuar os desejos e as intenções); e a elaboração de uma "nova linguagem do engano" (Young, 1995, p.52), que justificou a formação de uma classe médica especializada, investida da autoridade de reivindicar acesso ao conteúdo das memórias de seus portadores, avalizando a sua fidedignidade (Hacking, 1994; Herman, 1995; Fassin, Rechtman, 2009).

É, porém, apenas porque há um segredo que há a necessidade de preservá-lo. Em um *self* definido pelos atributos da memória, o esmaecimento ou o sobreinvestimento dos nexos associativos concentram o essencial para a localização de um fenômeno no campo da patologia ou da normalidade. Desde que Ribot (1883) estabeleceu a lei da regressão no campo científico da memória, passou-se a antever como a extraordinária revivificação de determinadas lembranças permanece a despeito da extinção aparente das condições que as propiciaram (Hacking, 1996). Simultaneamente, a sugestão pós-hipnótica, o "magnetismo animal" e outras técnicas de atuação sobre a memória revelavam a indisponibilidade dos traços mnêmicos e das experiências simbolizadas não acessíveis à recuperação imediata pelo *self* (Iserson, 2014). Haveria, então, um conjunto de nexos de memória cuja associabilidade estaria interditada à consciência, e de cuja existência teríamos notícia por manifestações misteriosas e eventualmente incontroláveis da mente humana (Janet, 1889; Freud, 2015, 2016, 2019). O surgimento da teoria do trauma deve a legitimação de seus postulados à indução sugestiva por meio da qual Charcot pôde inferir a indissociabilidade entre as crises histéricas e as reproduções das cenas traumáticas (Leys, 2000). Essa será uma concepção crítica para a especulação do fenômeno dissociativo, a partir do qual a operação simultânea de excomunhão e de manutenção do segredo patogênico termina por inaugurar uma nova etiologia das perturbações mentais.

Foi Pierre Janet quem expandiu o modelo do segredo patogênico a toda a extensão dos distúrbios histéricos (Young, 1995) – e nisso consiste a sua importância central para o campo dos estudos sobre o estresse traumático. Quando Charcot (1889) se referia à cerebração, mentação ou ideação subconsciente, ele não pensava no inconsciente dinâmico elaborado por Freud (2019) nem tinha interesse especial no conteúdo das ideias inconscientes, exceto quando elas serviam para explicar empiricamente o padrão neurológico e a localização dos sintomas histéricos como hemialgias ou paralisias funcionais (Young, 1995). Com Janet, diferentemente, a histeria se tornava uma janela para os mistérios da mente e do corpo (van der Kolk, 2016). Ao experimentar o que o autor classificara como "emoções veementes" (Janet, 1889), esses sujeitos ficariam destituídos de sua capacidade de sintetizar mentalmente as experiências assustadoras em seus esquemas cognitivos prévios, disso resultando que as memórias decorrentes não são integradas à consciência pessoal (van der Kolk, Hopper, Osterman, 2001). Pelo contrário, as experiências foram dissociadas da percepção consciente e do controle voluntário.

Nesse ponto convergem ainda as opiniões de Janet, Breuer e Freud: os sintomas histéricos emergem como vicissitudes de um processo psíquico carregado de afeto e impedido de acertar-se pela via normal que o conduziria à consciência e à motilidade (Freud, 2011). Desde o princípio, portanto, o pensamento do trauma psíquico pusera o elemento afetivo em primeiro plano, e a primeira formulação abrangente dos efeitos do trauma na mente foi baseada na noção de que a falha em integrar memórias traumáticas devido a extrema excitação emocional resulta nos sintomas do que hoje reconhecemos nos quadros dissociativos, os quais, muitas vezes, denominamos TEPT (van der Kolk, Hopper, Osterman, 2001; Breh, Seidler, 2007; Bryant, 2007).

Para isso, concorre o fato de que, no TEPT, diferentemente do que sucede em outras categorias nosográficas favoráveis ao enquadramento supostamente ateórico da APA, o aglomerado sindrômico não consegue definir suficientemente a entidade mórbida. Como resultado dessa especificidade, temos um transtorno mental cuja sintomatologia é tanto inespecífica quanto inconsistente. E, se o TEPT adquire relevância clínica cada vez maior desde a sua criação, isso se deve à formação de uma zona de raridade que alinhavou os seus sintomas a uma etiologia – o acontecimento traumático – e a um mecanismo patogênico – a memória traumática (Young, 2004). O TEPT burlou a própria racionalidade sindrômica do

DSM, pois foi edificado sobre uma manobra de exceção. Contrariamente a outros transtornos, teremos aqui uma categoria que não abdica de certa lógica intrínseca.

Essa coerência interna foi diversamente interpretada pelo campo do psicotraumatismo. Se para Bessel van der Kolk (2016) o resgate da obra de Janet (1889) estaria no centro da redescrição neuropsiquiátrica da psicopatologia traumática, para Allan Young (1995, 2004), o TEPT não poderia ser elucidado sem o retorno ao paradigma do sonho traumático na obra freudiana (Freud, 2010a). Em um caso, atrela-se a emoção veemente (Janet, 1889) ao fracasso na liquidação de experiências aterradoras – o que conduziria à dissociação das memórias traumáticas e a seu retorno sob a forma de experiências fragmentárias de revivescência; em outro, supõe-se uma sobrecarga da barreira cortical contra os estímulos que, combinada ao medo intenso e ao efeito de surpresa, seria capaz de ocasionar a efração característica da traumatização psíquica (Freud, 2010a). Em todo caso, sendo a teoria dissociativa de Janet ou a compulsão de repetição freudiana os modelos precursores da descrição neurobiológica do estresse traumático pela psiquiatria contemporânea, estamos ainda em um ponto de interseção: aquele que une a tradição psicodinâmica à tradição materialista. O trauma pode ser, portanto, uma superfície de contato entre a psicanálise, as teorias cognitivas e as neurociências.

## As duas memórias do trauma

A cena paradoxal de uma pessoa incapaz de realizar o recital narrativo de uma experiência que insiste em confrontá-la com uma situação adversa foi equacionada por Janet por meio da hipótese de que do trauma resultaria uma "fobia de memória" (Janet, 1919, 1925) correlata, capaz de obstruir a síntese de eventos traumáticos e separar as suas memórias correspondentes da consciência comum (Janet, 1889; van der Kolk, Hopper, Osterman, 2001). As ocasiões em que surgiram tais "representações" patogênicas, designadas por Freud e Breuer (2016) "traumas psíquicos" e os mecanismos pelos quais o afeto "entalado" flui para encontrar escoamento na inervação somática foram especulações comuns à tentativa de condicionar a liquidação dos resíduos traumáticos pela tradução da experiência em narrativa pessoal. Assim, poderiam ser direcionadas as reações de ansiedade que figuravam os traços de memória do trauma sob a fachada das percepções aterrorizantes, das preocupações obsessivas e as reexperiências somáticas (Janet, 1889).

O trauma equivale, portanto, ao momento preciso da fabricação do segredo patogênico, e é por essa razão que as pessoas dele se recordam paradoxalmente, ao mesmo tempo, muito e muito pouco (van der Kolk, 2016). Muito, quando a importância emocional de uma experiência codifica memórias detalhadas, persistentes e de extraordinária nitidez (van der Kolk, Fisler, 1995); muito pouco, porque as reminiscências não resolvidas do trauma não se oferecem ao conhecimento genuíno por parte de suas vítimas (Freud, 2011). Nesse ponto se separam os caminhos de Charcot, Janet, Breuer e Freud. Se desde Charcot se supunha que as ideias patogênicas manifestariam os seus efeitos traumáticos pela limitação das funções psíquicas decorrentes da instalação de um estado hipnoide, a partir de Freud (2016) a patogenia da ideia atrela-se ao conflito entre o seu conteúdo e as tendências dominantes da vida psíquica.

É assim que a ênfase do discurso sobre o traumatismo começa a recair sobre a fragmentação histérica da personalidade em consequência de uma situação de terror ou medo extremo – uma experiência que, por parecer abalar as capacidades cognitivo-perceptuais da vítima, tornaria a cena traumática indisponível para determinados tipos especiais de lembrança (Leys, 2000). A psique traumatizada foi conceituada como um aparato para registrar os golpes em um domínio extrínseco à consciência comum. E o hipnotismo foi utilizado como método psicoterapêutico para recuperar as lembranças esquecidas, dissociadas ou reprimidas, trazendo-as à consciência e à linguagem. Como expoente máximo da doutrina do esquecimento, a qual se aliam as modernas ciências da memória (Hacking, 1994), a dissociação representa um processo pelo qual determinadas funções mentais, geralmente integradas, compartimentam-se e passam a operar além do alcance da percepção consciente e da recuperação da memória (van der Hart, Horst, 1989).

Apesar das alegações de van der Hart e Horst (1989), a obra original de Janet alude muito pouco ao caráter traumático dos afetos e das emoções (Sanfelippo, Dagfal, 2020). A análise de neurocientistas como van der Kolk (2016), ao requisitar o pioneirismo da obra de Janet, de alguma forma antecipando os achados que um século depois as neurociências realizariam no domínio da memória traumática, omite que para o psicólogo francês a cisão da personalidade seria apenas a consequência de uma predisposição inata, uma fraqueza constitucional para a síntese dos elementos mentais devido ao estreitamento do campo da consciência. Reaplicado em uma roupagem epistemológica modernizada e com um verniz semântico contemporâneo, o modelo dissociativo teria provado que, apesar de passado um século, "Janet ainda fornece uma insuperável estrutura para integrar o conhecimento atual sobre os efeitos psicodinâmicos, cognitivos e biológicos da traumatização humana" (van der Kolk, Brown, van der Hart, 1989, p.365). Esse é mais um exemplo de como a interpretação presentista de argumentos e de fatos históricos pode servir ao reforço de teorias científicas contemporâneas não apenas pelo resgate, mas pela própria apropriação do pensamento de autores antepassados. É assim que a teoria neurocientífica do trauma, nomeando a teoria dissociativa de Janet como o seu marco inicial, atribui valor de verdade à sua interpretação e aos seus pressupostos.

Embora não seja o seu criador, Janet foi pioneiro em demonstrar que os fenômenos dissociativos desempenham papel central em respostas de estresse pós-traumático amplamente divergentes, por meio dos quais postulou o mecanismo de separação e de isolamento das impressões de memória que intuiu como mecanismos etológicos das perturbações histéricas (Janet, 1889). A ideia capaz de adoecer cresce, instala-se no pensamento, subtraindo ao indivíduo qualquer controle sobre o seu desenvolvimento, tanto porque ele a ignora quanto porque ela conquistou independência e existe em um campo de pensamento apartado e exclusivo (Janet, 1901). Dessa natureza seriam a fixação histérica das ideias (Janet, 1889) e os sintomas que, segundo a teoria freudiana, representam os símbolos mnêmicos e o retorno convertido de vivências traumáticas ainda atuantes (Freud, 2015). Fosse por meio da amnésia pós-traumática ou das reproduções intrusivas do choque emocional, as manifestações esperadas da codificação de experiências avassaladoras indicavam formatações específicas: a memória psicológica encontrando o exílio; a memória somática, a repetição pré-verbal insistente (Freud, 2010b; van der Kolk, 1996). Para aquela, não é concebível existir sem um vestígio simbolizado que a represente; para esta, a existência fica condicionada às presunções do medo, da dor e de um corpo simultaneamente histórico e material em que possa inscrever os seus efeitos transgeracionais. É impensável que uma tradição psicológica da investigação sobre o fenômeno traumático prescinda por completo da referência ao campo representacional ou que, atualmente, a sua contraparte somática recuse as pesquisas neurobiológicas e as suas respectivas tecnologias (Yehuda, McFarlane, Shalev, 1998; Leys, 2000, 2006; van der Kolk, 2000, 2002).

Quando Bessel van Der Kolk (2016) faz referência ao colapso funcional de regiões cerebrais devotadas a assegurar a sobrevivência, está se posicionando em um lado do debate da traumatização – aquele que apostará na capacidade de o evento traumático perturbar circuitos cerebrais e, ao menor sinal de perigo, induzir o organismo a secretar quantidades massivas de hormônios estressores (Everly, 1989; van der Kolk, Burbridge, Suzuki, 1997). A atuação da memória sobre o aparato cerebral precipitaria emoções indesejadas, sensações intensas e ações agressivas – respostas pós-traumáticas aparentemente incompreensíveis e esmagadoras. Quer seja pelo ângulo do cérebro desajustado, aferrado a respostas neurocomportamentais inadaptativas, quer pela perspectiva da subjetividade fraturada e quebradiça, o trauma será entendido como uma experiência que mergulha a vítima na cena traumática tão visceralmente a ponto de impedir uma mínima distância especular necessária ao conhecimento cognitivo do ocorrido (Leys, 2000).

A separação definitiva dessas matrizes da memória traumática acontecerá ao longo do século XX, após um processo de diferenciação que radicalizou a partilha do fenômeno da traumatização sob a forma de cosmologias do trauma progressivamente heterogêneas e, em larga medida, mesmo antagônicas. Por resistir ao conhecimento e à degradação pelo tempo, ou por encontrar meios expressivos os mais disruptivos, o trauma transporta as suas vítimas ao passado e as encerra no instante do seu acontecimento. Por essa razão, mais que uma patologia da memória, ele é, com efeito, uma patologia do tempo.

## Considerações finais

Tal qual o concebemos, o trauma é uma invenção histórica resultante da conversão da violência em dano traumático (Young, 1995). Ele teria nascido de duas experiências assíncronas: os palcos cirúrgicos do século XVII, onde foi reconhecido como lesão tissular, e as especulações do choque nervoso no século XIX, por meio do qual veio a ser a expressão de uma lesão molecular. As condições históricas para a psicologização do trauma, ocorrendo na interface entre os acidentes ferroviários do século XIX, o conhecimento científico da fisiologia e a apropriação médica da hipnose, apresentam as "trajetórias cruzadas" da heterogênea história da traumatização (Sanfelippo, 2018). As cadeias de desenvolvimento que daí se desdobram – envolvendo as interpretações somáticas e as análises psicológicas do traumatismo – produziram linhas de pesquisa específicas e eventualmente mesmo antagônicas: uma atribuindo o desfecho traumático ao efeito parasitário do segredo patogênico sobre aquele que o abriga; a outra, apostando nas perturbações fisiológicas, anatômicas e funcionais do corpo traumatizado.

Ainda que a extensão do trauma do corpo para o território da subjetividade sugira uma precedência histórica da traumatização mecânica, seguimos a hipótese de que esse deslocamento alude não a uma transposição da metáfora do trauma, mas, sim, a uma origem comum entre as suas variantes. Seja ela apresentada em sua versão somatizada ou psicológico-existencial, a memória traumática permanece irredutível ao vocabulário da descompensação metabólica do cérebro ou da fratura biográfica do *self*, pois em ambas as tradições científicas ela conserva a sua função epistemológica de elemento mediador entre uma violência e os seus produtos. Assim como o medo é a memória da dor, também o segredo será a memória do sofrimento; ambos são, afinal, a memória do trauma. Neste trabalho, procurou-se reconhecer quais condições históricas possibilitaram a emergência de uma metáfora do trauma que acolheu em seu domínio léxico toda a variedade da traumatização: daquela que incide sobre a transcendência da superfície subjetiva àquela que concerne à imanência da substância corporal.

Sanfelippo (2018) adota uma postura crítica em relação a essa interpretação. Segundo o autor, algumas das mais citadas historiografias do trauma incorrem em uma espécie de anacronismo, ao sugerir que elementos atualmente distintivos para a caracterização da experiência traumática, como o choque nervoso e as emoções, fossem igualmente relevantes no passado. Mesmo no trabalho de Erichsen (1867), os quadros clínicos descritos como *railway spine* não permitem uma completa equivalência com a histeria traumática ou o choque nervoso geral, descritos respectivamente por Charcot e Page. Por isso, a atribuição de um sentido atual a fatos e teorias pregressas se trataria de uma leitura "presentista" (Sanfelippo, 2018), um estilo argumentativo que explica mais a racionalidade científica contemporânea que, propriamente, o modo de organização de ideias e fatos pretéritos.[1]

Seja como fratura, efração psicológica ou memória coletiva, o conceito de trauma reflete as transformações na interpretação ocidental do sofrimento e do infortúnio, e indica a emergência de uma sensibilidade social contemporânea politicamente orientada para a reparação e para o testemunho (Fassin, Rechtman, 2009). Hoje, em sua máxima florescência, a pesquisa do trauma reveste o seu objeto com a densa tecnologia neurobiológica, prescrevendo, para ele, sistemas fisiológicos, marcadores metabólicos e uma causalidade fisicalista que secundarizou as antigas referências psicodinâmicas pelas quais antes se revelavam os seus mecanismos de instalação. Isso começa com o estabelecimento de uma espécie de racionalidade atomizada que, desde as hipóteses clínicas de Erichsen (1867) para a *railway spine*, viabilizou a liberação do pensamento etiopatogênico das referências imediatas e visíveis da lesão, localizando a etiologia traumática no território indefinido das contusões microscópicas e dos danos celulares. A perturbação traumática passava a responder por uma virtualidade permissiva a outras modalidades explicativas, e foi esse raciocínio patogênico aberto que possibilitou a incorporação da metáfora cirúrgica do trauma no seio de uma matriz psicodinâmica de seus fenômenos – a qual ela ajudou também a desenvolver. Curiosamente, a própria virtualização da unidade traumática, efetuada pela medicina do século XIX, propiciou as condições de emergência da formalização do trauma psíquico e de suas disciplinas correlatas.

A aposta em uma etiologia partilhada para a interpretação de condições pós-traumáticas, realizada por Young (1995, 1996a, 1996b), define, portanto, um posicionamento ético fundamental. Ela permite evitar abordagens reducionistas que se utilizem de determinismos, ora biológicos, ora psicodinâmicos, para explicar processos sociais e culturais complexos constituintes da vida humana, como a reexposição voluntária ao trauma e os efeitos transgeracionais da traumatização coletiva (Danieli, 1998; Goodman, 2013). Os trabalhos de Young (2000) – e mais recentemente de Ingold (2000) e Rose e Abi-Rached (2013) – podem auxiliar o reposicionamento do debate da traumatização, compromissando causalidades socioculturais, psicodinâmicas e neurobiológicas a uma mesma diretriz epistêmica. A elaboração de respostas culturalmente sensíveis e menos preocupadas em confinar os fenômenos da dor e do sofrimento a determinadas modalidades explicativas parece ser, portanto, uma resposta exequível à tendência neurocientífica contemporânea de reduzir a existência humana a propriedades cerebrais e a elementos neuroquímicos quantificáveis, assim como o século XIX fez com os produtos linguísticos do psiquismo (Mountcastle, 1998; Fuchs, 2018).

Dissemos que a virtualização da causalidade patogênica, com Erichsen (1867), permitiu a própria especulação do psicotraumatismo, e destacamos que a pesquisa científica do trauma estabeleceu historicamente uma profunda hierarquia entre seus paradigmas determinísticos, abrindo espaço para uma espécie de reducionismo materialista aplicado à interpretação de seus fenômenos. Isso foi o que permitiu a van der Kolk (2000) nomear de "redescoberta" o processo de resgate da atribuição causal de eventos externos já preconizada no "critério A" para o TEPT (American..., 1980). Se essa redescoberta do evento traumático como fator etiológico nos transtornos mentais tem apenas cerca de quarenta anos, é devido à sua desconsideração pelas causas psicodinâmicas como elemento etiológico do trauma. E van der Kolk recusa a causalidade subjetiva porque a condição de existência de qualquer traumatismo na tradição somática é que ele derive de um evento constituinte, sem o qual não haveria organismo traumatizado. Esse é o rito a ser respeitado, e o seu descumprimento pela tradição psicodinâmica – que apela à constituição e não ao agente causal – determinou que a interpretação neurobiológica, desde o século XX, proclamasse uma "idade das trevas", em que o trauma foi interpretado por referenciais pseudocientíficos e teve a sua compreensão, portanto, obscurecida. Assim, o trauma teria sido simplesmente ignorado desde a abertura etiológica do século XIX, para ser finalmente esclarecido, protegido, e rigorosamente valorizado pelas neurociências, após a segunda metade do século XX (van der Kolk, 2000). Essa espécie de imperialismo científico, pelo qual um determinado fenômeno passa a ter as suas descrições possíveis limitadas a um único prisma interpretativo, está no cerne da crítica de Ruth Leys (2000) para a apropriação do trauma pelas neurodisciplinas.

As duas memórias traumáticas – e as suas respectivas correntes de investigação – testemunham a duplicidade e a antinomia marcantes da história do pensamento sobre o traumatismo, como sugerem as complexas associações entre o trauma mecânico e o psicológico, entre o trauma único e o trauma repetido, entre o TEPT agudo e o TEPT crônico (Terr, 1984; Herman, 1995; Young, 1995; Fassin, Rechtman, 2009). Esse sistema difuso de oposições permanecerá transversal às discussões contemporâneas do trauma, sobretudo no que tange aos efeitos cumulativos e intergeracionais da traumatização (Marsella et al., 2008; Schwab, 2010). A compreensão das profundas transformações na economia moral do Ocidente é capital para reconhecer as inovações sociais que, se por um lado estimularam a invenção de novas áreas de conhecimento e práticas, por outro também propiciaram ramificações por meio da descoberta de novos pacientes e sujeitos. A medicina forense, as psiquiatrias de guerra, a psiquiatria militar, a psicanálise, a vitimologia psiquiátrica, a psiquiatria humanitária e a psicotraumatologia são personagens que protagonizam esses pontos de virada, só reconhecíveis se traduzidos segundo a dupla genealogia – científica e moral – que concebe o trauma no nível do debate teórico-prático, mas também se relaciona com as concepções sociais marcantes das mudanças de atitudes com respeito à autenticidade do sofrimento traumático (Fassin, Rechtman, 2009).

## NOTA

[1] Destacamos, no entanto, que a experiência do choque atomiza a causalidade patológica e inicia a distensão das possibilidades patogênicas para o trauma mecânico, incluindo nele um "elemento de incerteza" favorável à racionalidade psicodinâmica. A obra de Erichsen já atesta essa modificação.
